# Computed tomography-positive, SARS-CoV-2 RNA-negative symptomatic contacts of COVID-19 patients: what are their nature and implications?

**DOI:** 10.2217/fvl-2021-0205

**Published:** 2021-10-08

**Authors:** Lei Huang

**Affiliations:** ^1^Department of Oncology, Ruijin Hospital, Shanghai Jiao Tong University School of Medicine, 197 Ruijin Er Road, Shanghai, 200025, China

**Keywords:** COVID-19, CT positive, nature and implications, RNA negative, SARS-CoV-2, symptomatic contacts

COVID-19 has been spreading quickly around the globe, causing a pandemic. SARS-CoV-2 seems cunning and the full spectra of COVID-19 remains obscure [1]. Previously, we conducted a prospective population-based cohort study (Study 1) [2], and enrolled approximately 15,000 people undergoing SARS-CoV-2 RNA testing. We found that 0.2% of the people from eight clusters continued to test negative for SARS-CoV-2 RNA using common respiratory (swab and sputum), blood, urine and feces specimens and for all the other common respiratory pathogens from first sampling through to discharge. However, they had positive computed tomography (CT) findings, histories of close contact with laboratory-confirmed COVID-19 cases and symptoms. They had features more similar to confirmed COVID-19 cases than non-COVID-19 pneumonia cases. One of them transmitted COVID-19 to another person with no other exposures, who then became an asymptomatic patient [2].

The possible reasons why the CT-positive and virus RNA-negative (CPRN) cases kept testing negative for SARS-CoV-2 RNA could be as follows. The first possible explanation is the relatively low sensitivity of the virus RNA testing. However, in Study 1, respiratory specimens (both swab and sputum) were collected and processed by otolaryngologists with expertise in such procedures, and testing was centrally performed in the designated Center for Disease Control and Prevention and repeated with short intervals through to discharge, with authoritative guidelines carefully followed [3,4]. Cases with negative SARS-CoV-2 RNA findings received at least three rounds of quantitative RT-PCR tests, and a sensitivity of over 97% for at least three tests and the detection limit of 200 copies/ml, made the explanation of low-test sensitivity and low-sample virus load for the negative SARS-CoV-2 RNA findings less probable [5]. Furthermore, testing of the other specimens (blood, urine and feces) all showed negative findings. It is less possible that the tests themselves led to such a proportion of CPRN cases. The second possibility is that SARS-CoV-2 appeared in the specimens which were later examined only transiently (since exposure but before first specimen sampling); this could happen during the incubation period, when strong infectivity of the virus could already be present [1]. The patients might be majorly contagious during these periods. Afterward, all specimens continued to test negative for virus RNA through to discharge. If this most possible explanation does make sense, there could be a high risk that these patients transmitted SARS-CoV-2 to others before being quarantined, which markedly increases the difficulty and complexity of COVID-19 control. This possibility highlights the importance of careful, early and comprehensive monitoring and quarantine of all cases with any exposure history. Third, besides symptomatic laboratory-confirmed cases and asymptomatic carriers, both of whom have positive-virus RNA tests, such cases may represent a novel SARS-CoV-2 infection population. While infected with SARS-CoV-2, one patient had hardly detectable viruses in various samples. The CPRN cases had radiological, clinical and laboratory characteristics mostly similar with laboratory-confirmed COVID-19 patients, and might need to be managed in the same way as confirmed patients. Of course, there might be coexistence of both the last two explanations.

We further recommended a novel management pathway for this resource-limited pandemic era [2], based on careful, comprehensive, sequential evaluations of exposure history, symptoms and signs, pathogen or antibody test, and radiologic findings; the level of the medical resource and cost needed increases with the assessment sequence. Briefly, for the diagnosis of COVID-19, cases should first be assessed for exposure history. For those with or without unknown histories who are not in epidemic areas, if they do not have any COVID-19-relevant symptoms (e.g., fever and cough) [6], then they could be considered most likely uninfected; if they have any relevant symptoms, they should undergo a virus RNA test with or without an antibody test (for those without vaccination). Positive tests point to confirmed cases, all contacts of whom should be carefully traced, and for those with negative tests, searches for other pathogens should be done. For cases with positive exposure histories, virus RNA tests with or without antibody examinations should be performed regardless of symptoms, which will help to define cases. Cases with positive-virus RNA tests, with and without symptoms, are confirmed cases and asymptomatic carriers, respectively, all contacts of both of whom should be carefully traced after exposure. Asymptomatic cases with negative-virus RNA tests should undergo quarantine for at least 2 weeks with careful monitoring to confidently exclude SARS-CoV-2 infection. For symptomatic cases with continued negative-pathogen tests, further radiologic assessments should be performed to detect any insidious lesions. Based on Study 1 [2], those with positive-imaging findings should be managed as confirmed cases, all contacts of whom should be carefully traced; the others should be quarantined for at least 2 weeks with careful monitoring and with searches for other respiratory pathogens simultaneously. At the population level, a series of timely and strict interventions including active field investigation, case quarantine, contact tracing, education, centralization, closed management and boundary control as part of the One Health concept and approach could efficiently and effectively control COVID-19 outbreak in places with outbreaks originating from imported cases [6]. Precise and dynamic prevention and control measures should be implemented and based on characteristics including exposure history, age group, sex and phase of outbreak [6].

There are various modalities to diagnose COVID-19. Notably, the sensitivity of SARS-CoV-2 RNA tests using the quantitative RT-PCR method was only moderate according to a large population-based study, and had relatively high false-negative rates [7]. Single test of respiratory samples is most likely insufficient [8], and repeated sampling and tests could markedly increase the possibility of capturing COVID-19 infections, with the sensitivities of the first, second, third, and seventh tests being 72, 90, 97 and 100%, respectively [5]. Furthermore, throat swab samples might have lower viral loads compared with nasopharyngeal specimens, which had the best sensitivity for COVID-19 detection, especially at early stages of COVID-19 and in asymptomatic cases [9,10]. The noninvasive sputum or saliva samples also showed high sensitivity (~90%) and specificity (~100%) for COVID-19 detection, and had sensitivity similar to nasopharyngeal specimens [11,12]. For convenient samples, both swabs and sputum/saliva specimens are recommended. The sensitivity and specificity of rapid antigen test were as high as 98 and 100%, respectively, for detecting COVID-19 in cases with mild symptoms [13,14]. While serosurveillance using virus antibody tests could be important supplements to virus RNA tests to detect SARS-CoV-2 infection, the detectability depends largely on the severity and timing of COVID-19 infection and on the assay method used, which has varying reliability and requires critical validation, and they might be less relevant for diagnosis of ongoing COVID-19 infection nowadays with increasing popularization of SARS-CoV-2 vaccination [15–18]. Broncho-alveolar lavage specimens might further contribute to disclosing the nature of the CPRN cases; however, such a relatively invasive procedure might not be easily accepted by most of the patients with consent if not desperately needed. Furthermore, there exists a strong agreement between negative upper-respiratory swabs and broncho-alveolar lavage specimens, the latter of which has a limited role in COVID-19 diagnosis if upper-respiratory samples and thoracic radiologic findings are concordantly negative [19,20].

The laboratory findings in the CPRN cases in Study 1 were suggestive, but not conclusive, for COVID-19 infection, indicating possibly unique systemic anti-pathogen immune responses [2]. In another study of ours (Study 2) [21], longer intervals between illness onset and first blood sample collection were associated with a series of sequential laboratory value changes in blood routine, coagulation function, and biochemistry measures, and it is unclear whether the dynamic temporal evolutions are associated with the detectability of SARS-CoV-2, which warrants further investigation. Nonetheless, development of coagulation-fibrinolysis disorders, anemia, and malnutrition, which may be associated with various serious events in patients with COVID-19, should be closely monitored during disease progression and intervened when necessary.

While in Study 1 the duration from disease onset to discharge was markedly shorter in the CPRN cases with careful and active management than confirmed COVID-19 cases (median: 17 vs 27 days), they underwent continued quarantine at home or in hotels with careful monitoring after discharge, to ensure the elimination of the virus. For patients with confirmed COVID-19 in Study 2 [21], while the median duration of virus shedding was 11 days, 43% had a shedding duration of at least 2 weeks, and on admission they had more lymphocytes in peripheral blood and less often CRP levels above the upper threshold of the reference range compared with those with shedding duration of shorter than 2 weeks. The duration of SARS-CoV-2 shedding varied greatly, and on average a total period of 4 weeks since illness onset, after which the probability of virus shedding dropped to about 5%, is recommended for quarantine. While management of the CPRN cases was recommended to follow that of confirmed COVID-19 cases, it could possibly be more personalized.

The importance of clinical diagnosis cannot be underestimated in this pandemic era, and common SARS-CoV-2 RNA respiratory specimen (throat swab and sputum) tests by real-time RT-PCR assays, the widely accepted ‘golden standard’ for diagnosis of COVID-19, could be at times insufficient. Radiologic assessments might need to be further performed for SARS-CoV-2 RNA-negative symptomatic cases with positive COVID-19 exposure histories. To further disclose the nature of the SARS-CoV-2 RNA-negative (tested by common respiratory samples), CT-positive, symptomatic contacts of laboratory-confirmed COVID-19 patients, further investigations with some further procedures (e.g., examination of specific antibodies and/or broncho-alveolar lavage specimens) might be warranted. The clinical and epidemiological significances of such cases also need to be carefully reassessed with the emergence of several mutant SARS-CoV-2 variants.
